# Farnesiferol c induces apoptosis via regulation of L11 and c-Myc with combinational potential with anticancer drugs in non-small-cell lung cancers

**DOI:** 10.1038/srep26844

**Published:** 2016-05-27

**Authors:** Ji Hoon Jung, Moon Joon Kim, Hyemin Lee, Jihyun Lee, Jaekwang Kim, Hyun Joo Lee, Eun Ah Shin, Yoon Hyeon Kim, Bonglee Kim, Bum Sang Shim, Sung-Hoon Kim

**Affiliations:** 1 College of Korean Medicine, Kyung Hee University, Seoul, South Korea; 2 Graduate School of East-West Medical Science, Kyung Hee University, Yongin, South Korea

## Abstract

Though Farnesiferol c (FC) has been reported to have anti-angiogenic and antitumor activity, the underlying antitumor mechanism of FC still remains unclear. Thus, in the present study, we investigated the apoptotic mechanism of FC in human H1299 and H596 non-small lung cancer cells (NSCLCs). FC significantly showed cytotoxicity, increased sub-G1 accumulation, and attenuated the expression of Bcl-2, Bcl-xL, Survivin and procaspase 3 in H1299 and H596 cells. Furthermore, FC effectively suppressed the mRNA expression of G1 arrest related genes such as Cyclin D1, E2F1 transcription factor and CDC25A by RT-PCR. Interestingly, FC inhibited the expression of c-Myc, ribosomal protein L11 (L11) and nucleolin (NCL) in H1299 and H596 cells. Of note, silencing of L11 by siRNA transfection enhanced the expression of c-Myc through a negative feedback mechanism, while c-Myc knockdown downregulated L11 in H1299 cells. Additionally, combined treatment of FC and puromycin/doxorubicin promoted the activation of caspase 9/3, and attenuated the expression of c-Myc, Cyclin D1 and CDK4 in H1299 cells compared to single treatment. Taken together, our findings suggest that FC induces apoptosis and G1 arrest via regulation of ribosomal protein L11 and c-Myc and also enhances antitumor effect of puromycin or doxorubicin in NSCLCs.

Lung cancer is the leading cause of cancer related death all over the world and its main primary types are small lung cancer (10~15%) and non-small lung cancer (85~90%)^1^. In general, the treatment for lung cancer is surgery, chemotherapy, radiotherapy and targeted therapy mainly for EGFR or NF-κB^3^.

It was well documented that c-Myc is involved in proliferation, apoptosis, tumorigenesis, and cell cycle progression as one of the most frequently activated oncogene in human lung cancers^4^,^5^,^6^,^7^. Also, c-Myc was known to be regulated by ribosomal biogenesis related proteins, including L11, RPL5 and RPS14^8^,^9^,^10^. Especially, L11 was known to act as a novel c-Myc inhibitor^8^. Recently many natural compounds are attractive due to their cancer chemopreventive effects and potential to synergize with classical anticancer agents^11^,^12^,^13^.

Farnesiferol C (FC), a compound isolated from *Ferula assafoetida* L. has been reported to have cytotoxic^14^ and anti-angiogenic and antitumor effects^15^. Nonetheless, the underlying antitumor mechanism of FC was not fully understood so far. Thus, in the present study, the antitumor mechanism of FC was investigated in human H1299 and H596 non-small lung cancer cells (NSCLCs) in association with c-Myc and ribosomal protein L11 and also combinational potential of FC was examined with classical anticancer agents such as puromycin or doxorubicin in H1299 NSCLCs.

## Results

### FC induces cytotoxicity and apoptosis in non-small lung cancer cells

**Figure 1:**
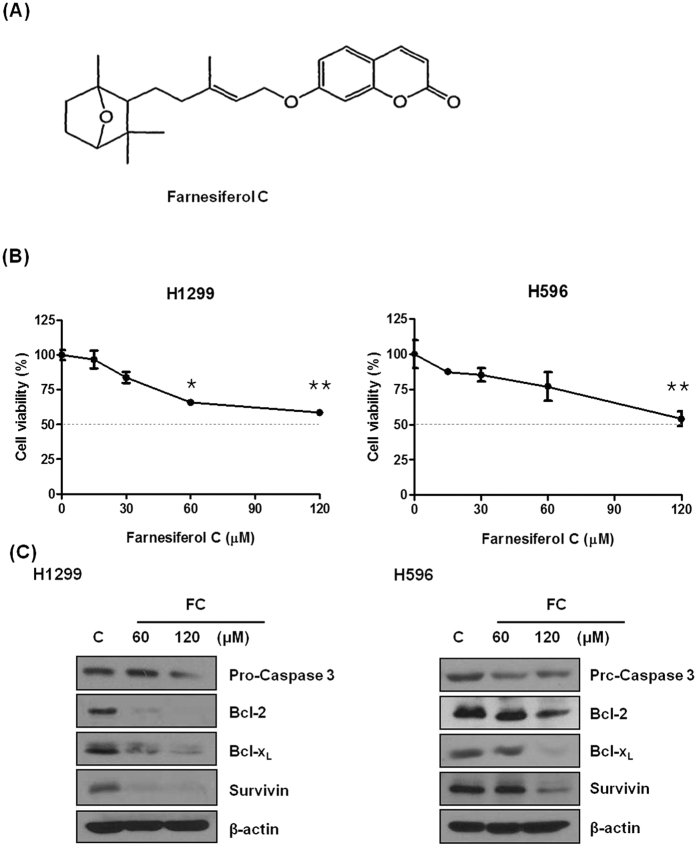
FC exerts cytotoxic and apoptotic activity in H1299 and H596 cells. (**A**) Chemical structure of FC. (**B**) Cytotoxicity of FC in H1299 and H596 cells by MTT assay. Data represent means ± SD. *p < 0.05. **p < 0.01 vs untreated control. (**C**) Effect of FC on procaspase 3, Bcl-2, Bcl-xL, and Survivin in H1299 and H596 cells by Western blotting.

Cytotoxicity of FC was evaluated in H1299 and H596 cells using MTT assay. As shown in Fig. 1B, FC significantly decreased the viability of H1299 and H596 cells. To examine whether or not the cytotoxic effect of FC is associated with apoptosis, cells were treated with various concentrations of FC in H596 and H1299 cells for 24 h. As shown in Fig. 1C, FC attenuated the expression of pro-caspase3, Bcl-2, Bcl-x_L_ and Survivin in H596 and H1299 cells.

### FC increases sub G1 population and attenuates the expression of Cyclin D1 and CDK4

**Figure 2:**
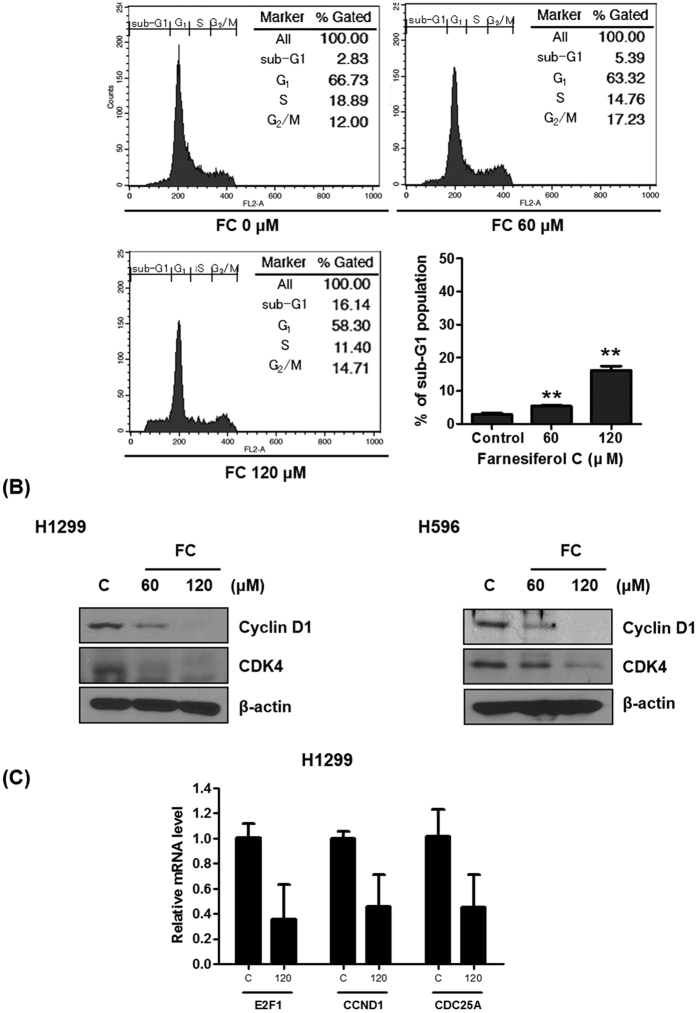
FC induces accumulation of subG1 phase in H1299 cells. (**A**) H1299 cells were treated with FC (0, 60, or 120 μM) for 24 h. Thereafter the cells were washed, fixed, stained with PI, and analyzed for DNA contents using a flow cytometry. **p < 0.01 vs untreated control. (**B**) Effect of FC on Cyclin D1 and CDK4 in H1299 and H596 cells by Western blotting. (**C**) Effect of FC on E2F1, CCND1, and CDC25A in H1299 cells by qRT-PCR.

To confirm the effect of FC on cell death and cell cycle arrest in cancer cells, H1299 cells were treated with various concentrations of FC for 24 h. The cells were stained with propidium iodide, and cell-cycle was analyzed by flow cytometry. As shown in Fig. 2A, FC increased sub G_1_ population up to 5.39% and 16.14% by at the concentrations of 60 μM and 120 μM, respectively, compared to untreated control (2.83%) in H1299 cells. Consistently, FC treatment significantly decreased the expression of Cyclin D1 and CDK4 in H1299 and H596 cells, since Cyclin D1 binds and activates Cyclin-dependent kinases 4 and 6 (CDK4 and CDK6), which regulate G1/S transition^16^ (Fig. 2B). Also, mRNA levels of E2F1, CCND1, and transcription factor and cell division cycle 25 homolog A (CDC25A), which are related to G_1_ phase^17^, were dramatically suppressed by FC treatment compared to untreated control (Fig. 2C).

### Regulation of L11 and c-Myc is critically involved in apoptosis induced by FC in H1299 cells

**Figure 3:**
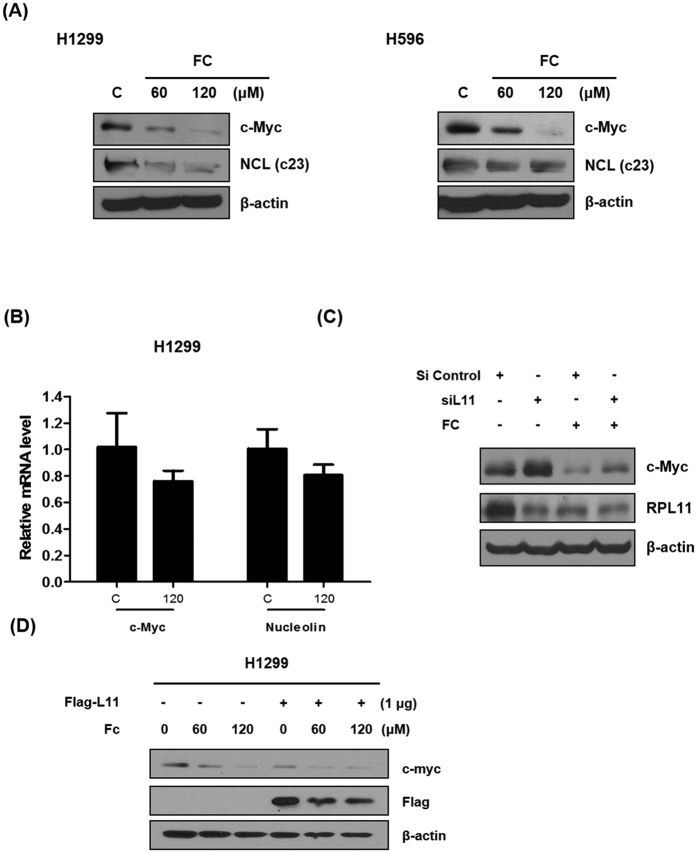
FC regulates c-Myc and L11 through a negative feedback mechanism. (**A**) FC downregulates c-Myc and NCL (C23) in H1299 and H596 cells by Western blotting. (**B**) FC attenuates mRNA expression of c-Myc and NCL in H1299 cells. H1299 cells were treated with FC for 24 h. RNA was isolated and analsis was performed to detect mRNA of c-Myc and NCL by qRT-PCR. (**C**) L11 knockdown activates c-Myc expression in H1299 cells. H1299 cells were transfected with L11 siRNA oligonucleotide (40 nM) for 48 h and then treated with FC for 24 h. Cells were prepared and subjected to Western blotting for c-Myc and L11. (**D**) FC downregulates c-Myc and L11 in Flag tagged L11 overexpression vector transfected H1299 cells. H1299 cells were transfected with Flag tagged L11 vector (1 μg) for 48 h and then treated with FC (0, 60, or 120 μM) for 24 h. Cells were prepared and subjected to Western blotting for c-Myc and L11.

**Figure 4:**
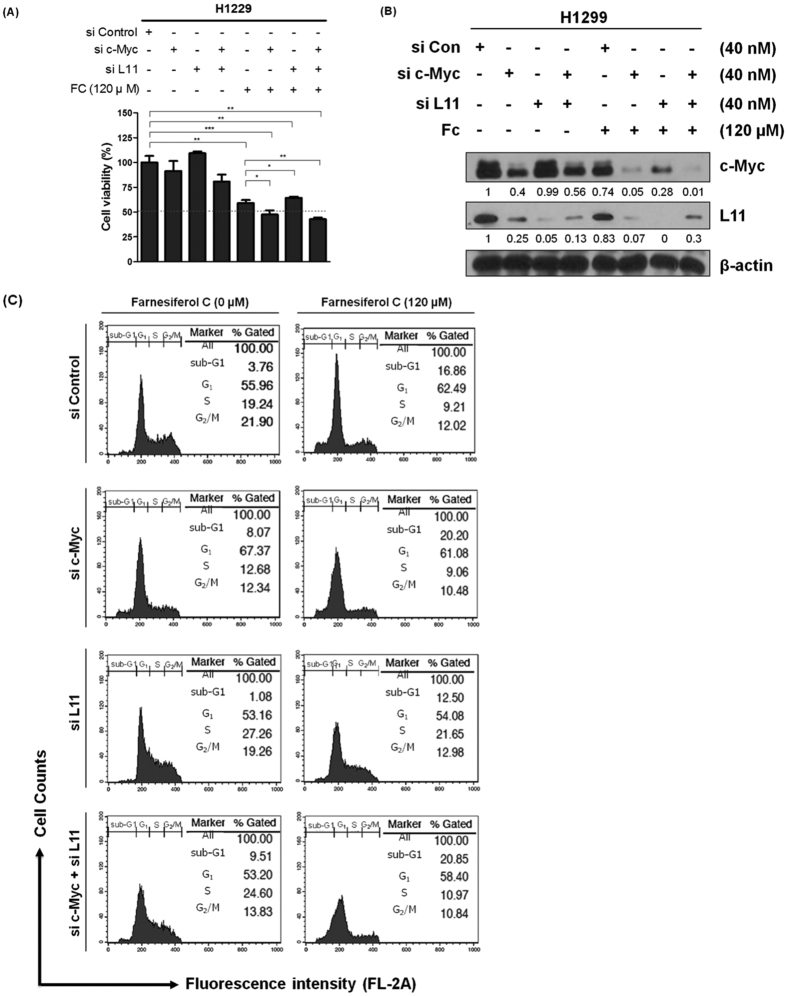
Regulation of L11 and c-Myc is critically involved in apoptosis induced by FC in H1299 cells. (**A**) Effect of c-Myc and/or L11 depletion by siRNA on the viability of H1299 cells by MTT assay. Data represent means ± SD. *p < 0.05, **p < 0.001, ***p < 000.1 (**B**) Effect of c-Myc and/or L11 depletion by siRNA on the expression of c-Myc and L11 in H1299 cells. H1299 cells were transfected with c-Myc and/or L11 siRNA oligonucleotide (40 nM) for 48 h and then treated with FC for 24 h. Cells were prepared and subjected to Western blotting for c-Myc and L11. (**C**) Effect of c-Myc and/or L11 depletion by siRNA on sub G1 accumulation in H1299 cells. H1299 cells were treated with FC (120 μM) for 24 h. Then the cells were washed, fixed, stained with PI, and analyzed for DNA contents using a flow cytometry.

Apoptosis is regulated by various proteins such as c-Myc, caspase3, and bcl-2^18^,^19^. FC dose-dependently suppressed the protein expression of c-Myc and its downstream NCL (nucleolin; c23) in H1299 cells, while it attenuated the expression of c-Myc alone in H596 cells (Fig. 3A). In contrast, FC attenuated the mRNA expression of c-Myc and NCL by only ~25% in H1299 cells (Fig. 3B). To validate the regulation of L11 and/or c-Myc in apoptosis induced by FC treatment, we performed siRNA transfection assay for L11 and/or c-Myc. Silencing of c-Myc promoted the ability of FC to exert cytotoxicity and sub G1 accumulation, while L11 knockdown suppressed the antitumor activity of FC in H1299 cells (Fig. 4A,C). In contrast, L11 overexpression using Flag L11 plasmid attenuated the expression of c-Myc in a concentration dependent fashion in H1299 cells. Furthermore, silencing of both L11 and c-Myc promoted cytotoxicity and sub G1 accumulation in H1299 cells compared to single treatment. Unexpectedly, Western blotting revealed that FC downregulated the expression of not only c-Myc but also L11 compared to untreated control in H1299 cells as shown in Figs 3C and 4B. Furthermore, overexpression of L11 using Flag tagged L11 expression vector suppressed the expression of c-Myc in a concentration dependent fashion in H1299 cells (Fig. 3D) and also FC attenuated the expression of c-Myc and overexpressed L11 in H1299 cells. Of note, c-Myc knockdown enhanced downregulation of L11, whereas silencing of L11 induced upregulation of c-Myc in H1299 cells (Fig. 4B).

### FC promotes puromycin or doxorubicin-induced apoptosis in H1299 cells

**Figure 5:**
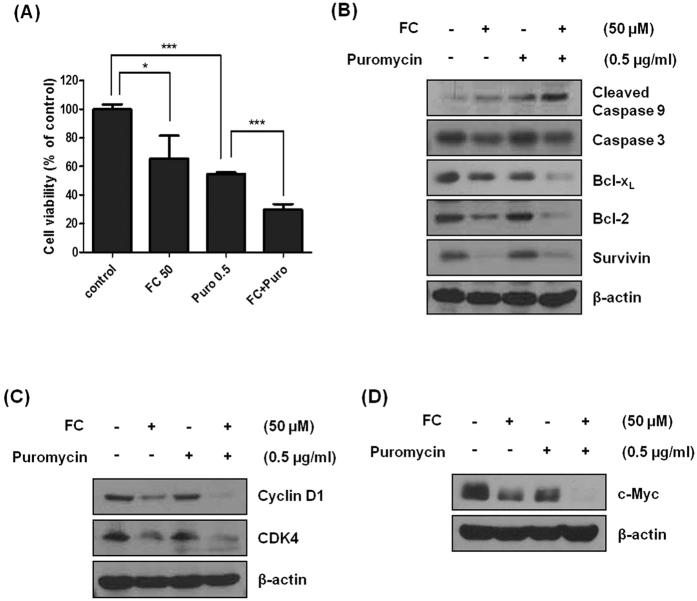
Combination effect of FC and puromycin on cytotoxicity and apoptosis or G1 phase related proteins in H1299 cells. (**A**) FC significantly enhanced cytotoxicity of puromycin in H1299 cells. The cells were treated with FC (50 μM) and/or puromycin (0.5 μg/ml) for 24 h. Cytotoxicity of FC and puromycin in H1299 and H596 cells by MTT assay. Data represent means ± SD. *p < 0.05, ***p < 000.1 vs untreated control. (**B**) Combinational effect of FC and puromycin on caspase9/3, Bcl-2, Bcl-x_L_ and Survivin in H1299 cells by Western blotting. (**C**) Combinational effect of FC and puromycin on cyclin D1, CDK4 and c-Myc in H1299 cells by Western blotting.

**Figure 6:**
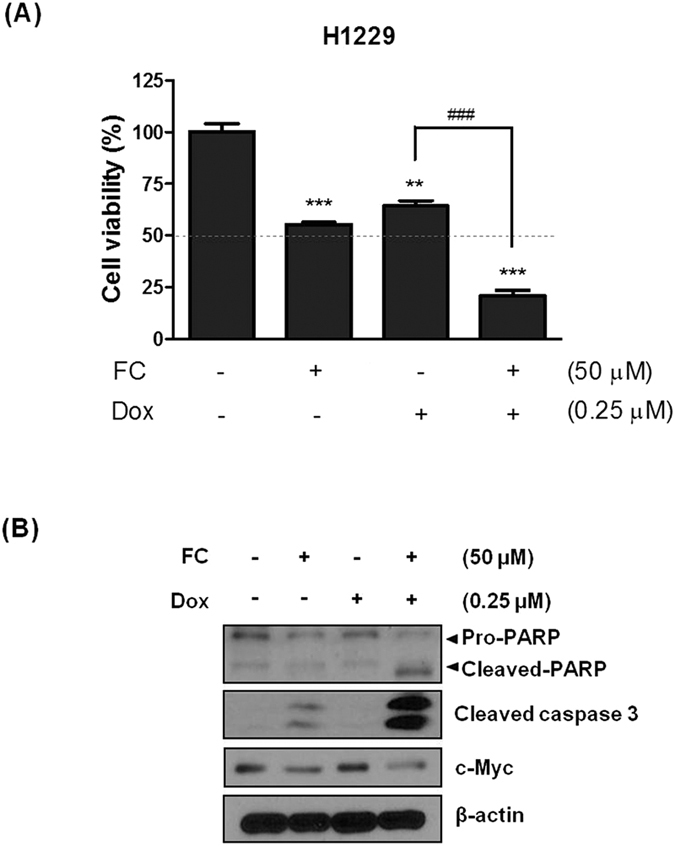
Combination effect of FC and doxorubicin on cytotoxicity and apoptosis related proteins in H1299 cells. (**A**) Effect of FC or doxorubicin on cytotoxicity in H1299 cells. The cells were treated with FC (0~120 μM) and/or doxorubicin (0~4 μM) for 24 h. Cytotoxicity of FC and/or doxorubicin in H1299 cells by MTT assay. Data represent means ± SD. (**B**) Combinational effect of FC (50 μM) and doxorubicin (0.25 μM) on cyctotoxicity in H1299 cells by MTT assay. **p < 0.01, ***p < 000.1 vs untreated control. (**C**) Combinational effect of FC (50 μM) and doxorubicin (0.25 μM) on cleaved caspase 9/3 and c-Myc in H1299 cells by Western blotting.

Previous studies suggested that puromycin enhanced apoptosis with melatonin in human leukemia HL-60 cells^20^. In the same line, to evaluate the combinational potential of FC with puromycin or doxorubicin, MTT assay and Western blotting were performed in H1299 cells. FC enhanced weak cytotoxicity of puromycin at a nontoxic concentration of 0.5 μg/ml in H1299 cells by combination treatment (Fig. 5A) Consistently, Western blotting showed that FC promoted weak apoptotic potential of puromycin to cleave caspase 9 and attenuate the expression of pro-caspase3, Bcl-2, Bcl-x_L,_ Survivin Cyclin D1, CDK4 and c-Myc in H1299 cells (Fig. 5B–D). In addition, we used 0.25 μM of doxorubicin for combination therapy with FC, since Rathos *et al*. reported the IC50 of doxorubicin is 0.23 μM in H1299 cells^21^. FC enhanced weak cytotoxicity and apoptotic activity of doxorubicin via the cleavage of caspase 3 and reduced expression of c-Myc in H1299 cells (Fig. 6A,B).

## Discussion

Lung cancer still remains the leading cause of cancer-related mortality in the world. The majority of the lung cancer patients are the non-small cell (NSCLC) subtype^22^. Since, identification of epidermal growth factor receptor (EGFR) mutations and anaplastic lymphoma kinase (ALK) rearrangements in NSCLCs promoted targeted therapies^3^,^23^, several phytochemicals are attractive due to less toxicity and synergistic potential with classical anticancer agents^12^,^24^,^25^,^26^,^27^,^28^,^29^.

Previously our group reported that FC had anti-angiogenic and antitumor activity targeting VEGFR1 or VEGFR2 signaling cascades. Nevertheless, the underlying antitumor mechanism of FC is not still unclear. Thus, in the current study, molecular antitumor mechanism of FC was examined in non-small lung cancer cells.

FC significantly exerted cytotoxicity, increased sub-G1 accumulation for apoptotic portion and attenuated the expression of survival genes such as Bcl-2, Bcl-x_L_, Survivin and pro-caspase 3 in H1299 and H596 cells, strongly implying the apoptotic activity of FC.

Cell cycle arrest is known as a stopping point in the cell cycle transition such as G0, G1, S and G2/M phases for cell duplication and division. Binding of Cyclin D1 and CDK4 or CDK6 was well known to trigger the transition from G1 to S phase^17^,^28^,^32^. Thus, cell cycle regulation is regarded as a good target for cancer therapy^30^. In the current study, FC effectively induced G1 arrest by suppressing G1/S transition phase related mRNA such as Cyclin D1, E2F1 and CDC25A and significantly decreased the protein expression of Cyclin D1 and CDK4 in H1299 and H596 cells, indicating G1 phase arrest by FC treatment.

The c-Myc is a multifunctional oncogene involved in cell growth, proliferation, tumorigenesis, and so is frequently upregulated in various types of cancer cells^31^. One of the key biological functions of c-Myc is to promote cell-cycle progression in several cancers^32^. After serum stimulation, c-Myc is induced at mRNA and protein levels and the cells enter the G1 phase of the cell cycle to promote biological processes including proliferation or apoptosis^33^. Interestingly, FC inhibited the expression of c-Myc and its downstream NCL at mRNA and protein levels in H1299 and H596 cells, demonstrating the role of c-Myc inhibition in FC-induced apoptosis and G1 arrest in NSCLCs. Also, it is noteworthy that FC reduced mRNA level of c-Myc by about 25%, while the protein level c-Myc was almost disappeared by FC, implying that FC works on c-Myc rather at posttranscriptional level, though further study is required in the near future.

Ribosomes are essential components of the protein synthesis machinery and ribosomal proteins play a critical role in cell proliferation, differentiation, apoptosis, DNA repair, and other cellular processes^10^. Among many ribosomal proteins, ribosomal protein L5 or L11 binds to c-Myc^8^ and also activates TAp73 by overcoming MDM2 inhibition^9^, consequently leading to inhibition of c-Myc activity. Here, silencing of c-Myc promoted the ability of FC to exert cytotoxicity and sub G1 accumulation in H1299 cells, whereas L11 knockdown abrogated the antitumor activity of FC in H1299 cells. Of note, though FC downregulated the expression of not only c-Myc but also L11 compared to untreated control in H1299 cells, c-Myc knockdown enhanced the downregulation of L11 and silencing of L11 induced upregulation of c-Myc in H1299 cells, indicating the possible feedback regulation of c-Myc by L11. These results were supported by previous report^34^ by Dai *et al*. that reduction of L11 by siRNA increases c-Myc levels through a negative feedback mechanism, since L11 directly binds to the Myc box II (MB II) for c-Myc-enhanced ribosomal biogenesis.

Recently combination therapy of anticancer agents and natural compounds are on the spotlight for reducing side effect and enhancing antitumor activity^35^,^36^. Here, combined treatment of FC and puromycin/doxorubicin promoted the activation of caspase 9/3, and attenuated the expression of c-Myc, Cyclin D1 and CDK4 in H1299 cells compared to single treatment, demonstrating the potential of using FC for combination therapy with classical anticancer agents such as puromycin or doxorubicin.

In summary, FC significantly showed cytotoxicity, increased sub-G1 accumulation, attenuated the expression of Bcl-2, Bcl-x_L_, Survivin and procaspase 3, suppressed the mRNA expression of G1 arrest related genes such as Cyclin D1, E2F1 and CDC25A, reduced the expression of c-Myc and NCL at mRNA and protein levels in H1299 and H596 cells. Interestingly, knockdown of L11 enhanced the expression of c-Myc through a negative feedback mechanism in H1299 cells. Furthermore, combined treatment of FC and puromycin/doxorubicin promoted the activation of caspase 9/3, and attenuated the expression of c-Myc, Cyclin D1 and CDK4 in H1299 cells. Overall, our findings suggest that FC induces apoptosis and G1 arrest via regulation of L11 and c-Myc with combinational potential with puromycin or doxorubicin in NSCLCs.

## Methods

### Reagents

FC was extracted from *Ferula assafoetida* as previously described^37^. FC (C_24_H_30_O_4_, MW 382; Fig. 1A) with the purity (over 98% by HPLC) was dissolved in DMSO, stored as small aliquots at −80 °C, and then diluted for cell culture study.

### Cell culture

Human lung cancer cell lines H1299 and H596, which are non-small lung cancer cell lines were obtained from American Type Culture Collection, and maintained in RPMI1640 medium supplemented with 10% fetal bovine serum (Gibco, Carlsbad, CA, USA), 2 μM L-glutamine and penicillin/streptomycin (WelGene, South Korea).

### Cytotoxicity assay

Cytotoxic effect of FC was evaluated by 3-(4,5-dimethylthiazol-2-yl)-2,5-diphenyl tetrazolium bromide (MTT) assay. Cells were seeded onto 96-well microplates at a density of 1 × 10^4^ cells/well and treated with various concentrations of FC (0, 10, 20, 40, 80 or 160 μM) for 24 h. The cells were incubated with MTT solution (1 mg/ml) (Sigma Chemical Co., USA) for 2 h and DMSO for 2 h. Optical density (OD) was measured using a microplate reader (Molecular Devices Co., Sunnyvale, CA) at 570 nm. Cell viability was calculated as a percentage of viable cells in FC-treated group *versus* untreated control by the following equation. Cell viability (%) = [OD (FC) − OD (Blank)]/[OD (Control) − OD (Blank)] × 100.

### Cell cycle analysis

Cell cycle analysis was performed by propidium iodide (PI) staining. Cells were fixed in 75% ethanol, incubated with 0.1% RNaseA in PBS at 37 °C for 30 min and resuspended in PBS containing 25 μg/ml PI for 30 min at room temperature. The stained cells were analyzed for DNA content by FACSCalibur (Becton Dickinson, Franklin Lakes, NJ, USA) using Cell Quest program (Becton Dickinson, Franklin Lakes, NJ, USA).

### Western blot analysis

Cells were lysed in RIPA buffer (50 mM Tris-HCl, pH 7.4, 150 mM NaCl, 1% NP-40, 0.25% sodium deoxycholic acid, 1 M EDTA, 1 mM Na_3_VO_4_, 1 mM NaF and protease inhibitors cocktail). Protein samples were quantified by using a Bio-Rad DC protein assay kit II (Bio-Rad, Hercules, CA), separated by electrophoresis on 8 to 15% SDS-PAGE gel and electro transferred onto a Hybond ECL transfer membrane (Amersham Pharmacia, Piscataway, NJ). After blocking with 3–5% nonfat skim milk, the membrane was probed with antibodies for cleaved Caspase 9, Bcl-x_L_, (Cell signaling Technology, Danvers, MA), c-Myc, L11 (Abcam, Cambridge, United Kingdom), Cyclin D1, CDK4, Bcl-2, Caspase 3, NCL, Survivin (Santa Cruz Biotechnologies, Santa Cruz, CA, USA), and β-actin (Sigma Aldrich Co., St. Louis, MO) followed by exposing to horseradish peroxidase (HRP)-conjugated secondary anti-mouse or rabbit antibodies (AbDserotec, kidlington, UK). Protein expression was determined by using enhanced chemiluminescence (ECL) system (Amersham Pharmacia, Piscataway, NJ, USA).

### Real-time quantitative RT-PCR analysis (RT-qPCR)

Total RNA was isolated by using RNeasy mini kit (Qiagen, Valencia, CA) according to the manufacturer’s instructions and reverse transcribed using M-MLV reverse transcriptase (Promega, Madison, WI). The cDNA was amplified by PCR using the synthesized specific primers as follows (Bioneer, Daejeon, Korea): *c-Myc* forward 5′-CCACCAGCAGCGACTCTGA-3′ and reverse 5′-GCAGAAGGTGATCCAGACTC-3′; *E2F1* forward 5′-TCCAAGAACCACATCCAGTG-3′ and reverse 5′-CTGGGTCAACCCCTCAAG-3′; *CCND1* forward 5′- GAAGATCGTCGCCACCTG-3′ and reverse 5′-GACCTCCTCCTCGCACTTCT-3′; *CDC25A* forward 5′- ATCTCTTCACACAGAGGCAGAA-3′ and reverse 5′- CCCTGGTTCACTGCTATCTCTT-3′; *NCL* forward 5′-GTGGTGGACGGTGTTCACTT-3′ and reverse 5′-GCCACGGCCAGCACATCAT-3′; *GAPDH* forward 5′-CTGCACCACCAACTGCTTAG-3′ and reverse 5′-AGGTCCACCACTGACACGTT-3′.

RT-qPCR was operated with the light cycler TM instrument (Roche Applied Sciences, Indianapolis, IN) according to the manufacturer’s protocol. The mRNA level of GAPDH was used to normalize the expression of genes of interest.

### RNA transfection assay

Cells were transiently transfected with a validated scrambled control small interfering (si)RNA, or siRNA specifically for L11 or c-Myc (Santa Cruz Biotechnology, Santa Cruz, CA) or Flag–tagged L11 overexpression vector kindly provided by Prof. Hua Lu (School of Medicine, Oregon Health and Science University, USA) by using Interferin^TM^ transfection reagent (Polyplus-transfection Inc., New York, NY). Briefly, the mixture of siRNA or Flag–tagged L11 overexpression vector with Interferin^TM^ transfection reagent was incubated for 10 min, added to each well of the cells (siRNA final concentration = 40 nM) and incubated at 37 °C for 48 h before drug treatment.

### Statistical analyses

Data were presented as means ± standard deviation (SD) of three or more replicates. Statistical significance was verified by Student’s *t-test* using Sigma plot software (System Software Inc., San Jose, CA, USA).

## Additional Information

**How to cite this article**: Jung, J. H. *et al*. Farnesiferol c induces apoptosis via regulation of L11 and c-Myc with combinational potential with anticancer drugs in non-small-cell lung cancers. *Sci. Rep*. **6**, 26844; doi: 10.1038/srep26844 (2016).

## References

[b1] Leung C. C. . Year in review 2013: Lung cancer, respiratory infections, tuberculosis, cystic fibrosis, pleural diseases, bronchoscopic intervention and imaging. Respirology 19(3), 448–460 (2014). 24708034 10.1111/resp.12250 PMC4151183

[b2] Amorin K. E. Lung cancer: a review of current knowledge, diagnostic methods and therapeutic perspectives. Rev. Peru. Med. Exp. Salud Publica 30(1), 85–92 (2013). 23612819 10.1590/s1726-46342013000100017

[b3] Abidin A. Z. . Targeted therapies in small cell lung cancer: a review. Ther. Adv. Med. Oncol. 2(1), 25–37 (2010). 21789124 10.1177/1758834009356014 PMC3126006

[b4] Grandori C. . c-Myc binds to human ribosomal DNA and stimulates transcription of rRNA genes by RNA polymerase I. Nat. Cell. Biol. 7(3), 311–U121 (2005). 15723054 10.1038/ncb1224

[b5] Pelengaris S., Khan M. & Evan G. c-MYC: More than just a matter of life and death. Nat. Rev. Cancer 2(10), 764–776 (2002). 12360279 10.1038/nrc904

[b6] Tsai L. H. . The MZF1/c-MYC axis mediates lung adenocarcinoma progression caused by wild-type lkb1 loss. Oncogene 34(13), 1641–9 (2015). 24793789 10.1038/onc.2014.118

[b7] Ciribilli Y. . Decoding c-Myc networks of cell cycle and apoptosis regulated genes in a transgenic mouse model of papillary lung adenocarcinomas. Oncotarget 6(31), 31569–92 (2015). 26427040 10.18632/oncotarget.5035 PMC4741625

[b8] Liao J. M., Zhou X., Gatignol A. & Lu H. Ribosomal proteins L5 and L11 co-operatively inactivate c-Myc via RNA-induced silencing complex. Oncogene 33(41), 4916–4923 (2014). 24141778 10.1038/onc.2013.430 PMC4026346

[b9] Zhou X. . Ribosomal proteins L11 and L5 activate TAp73 by overcoming MDM2 inhibition. Cell Death Differ. 22(5), 755–66 (2014). 25301064 10.1038/cdd.2014.167 PMC4392073

[b10] Kim T. H., Leslie P. & Zhang Y. Ribosomal proteins as unrevealed caretakers for cellular stress and genomic instability. Oncotarget 5(4), 860–871 (2014). 24658219 10.18632/oncotarget.1784 PMC4011588

[b11] Jung M. H., Lee S. H., Ahn E. M. & Lee Y. M. Decursin and decursinol angelate inhibit VEGF-induced angiogenesis via suppression of the VEGFR-2-signaling pathway. Carcinogenesis 30(4), 655–661 (2009). 19228635 10.1093/carcin/bgp039

[b12] Liu Z. . Ethoxysanguinarine Induces Inhibitory Effects and Downregulates CIP2A in Lung Cancer Cells. ACS Med. Chem. Lett. 5(2), 113–118 (2014). 24900782 10.1021/ml400341k PMC4027744

[b13] Bi X. . Anti-tumor activity of three ginsenoside derivatives in lung cancer is associated with Wnt/beta-catenin signaling inhibition. Eur. J. Pharmacol. 742, 145–52 (2014). 25199964 10.1016/j.ejphar.2014.08.032

[b14] Valiahdi S. M., Iranshahi M. & Sahebkar A. Cytotoxic activities of phytochemicals from Ferula species. Daru 21(1), 39 (2013). 23701832 10.1186/2008-2231-21-39 PMC3671137

[b15] Lee J. H. . Herbal compound farnesiferol C exerts antiangiogenic and antitumor activity and targets multiple aspects of VEGFR1 (Flt1) or VEGFR2 (Flk1) signaling cascades. Mol. Cancer Ther. 9(2), 389–399 (2010). 20103598 10.1158/1535-7163.MCT-09-0775

[b16] Georgantas R. W. . MicroRNA-206 induces G1 arrest in melanoma by inhibition of CDK4 and Cyclin D. Pigment Cell Melanoma Res. 27(2), 275–286 (2014). 24289491 10.1111/pcmr.12200

[b17] Hochegger H., Takeda S. & Hunt T. Cyclin-dependent kinases and cell-cycle transitions: does one fit all? Nat. Rev. Mol. Cell Biol. 9(11), 910–916 (2008). 18813291 10.1038/nrm2510

[b18] BCL2 Tomita N. and MYC dual-hit lymphoma/leukemia. J. Clin. Exp. Hematop. 51(1), 7–12 (2011). 21628855 10.3960/jslrt.51.7

[b19] Cinar M. . Concurrent inhibition of MYC and BCL2 is a potentially effective treatment strategy for double hit and triple hit B-cell lymphomas. Leuk. Res. 39(7), 730–738 (2015). 25916698 10.1016/j.leukres.2015.04.003

[b20] Koh W. . Melatonin promotes puromycin-induced apoptosis with activation of caspase-3 and 5′-adenosine monophosphate-activated kinase-alpha in human leukemia HL-60 cells. J. Pineal Res. 50(4), 367–73 (2011). 21244482 10.1111/j.1600-079X.2010.00852.x

[b21] Rathos M. J. . Potentiation of *in vitro* and *in vivo* antitumor efficacy of doxorubicin by cyclin-dependent kinase inhibitor P276-00 in human non-small cell lung cancer cells. BMC Cancer 13, 29 (2013). 23343191 10.1186/1471-2407-13-29 PMC3635914

[b22] Siegel R., Ma J., Zou Z. & Jemal A. Cancer statistics, 2014. CA Cancer J. Clin. 64(1), 9–29 (2014). 24399786 10.3322/caac.21208

[b23] Stinchcombe T. E. Novel agents in development for advanced non-small cell lung cancer. Ther. Adv. Med. Oncol. 6(5), 240–253 (2014). 25342991 10.1177/1758834014532510 PMC4206610

[b24] Chae S. . Effect of compound K, a metabolite of ginseng saponin, combined with gamma-ray radiation in human lung cancer cells *in vitro* and *in vivo*. J. Agric. Food Chem. 57(13), 5777–5782 (2009). 19526988 10.1021/jf900331g

[b25] Gomathinayagam R. . Anticancer mechanism of plumbagin, a natural compound, on non-small cell lung cancer cells. Anticancer. Res. 28(2A), 785–792 (2008). 18507021

[b26] Singhal S. S. . Novel compound 1,3-bis (3,5-dichlorophenyl) urea inhibits lung cancer progression. Biochem. Pharmacol. 86(12), 1664–1672 (2013). 24099794 10.1016/j.bcp.2013.09.022 PMC4186798

[b27] Song Y. . A novel small-molecule compound diaporine A inhibits non-small cell lung cancer growth by regulating miR-99a/mTOR signaling. Cancer Biol. Ther. 15(10), 1423–1430 (2014). 25046358 10.4161/cbt.29925 PMC4130735

[b28] Calbo J. . G1 cyclin/cyclin-dependent kinase-coordinated phosphorylation of endogenous pocket proteins differentially regulates their interactions with E2F4 and E2F1 and gene expression. J. Biol. Chem. 277(52), 50263–50274 (2002). 12401786 10.1074/jbc.M209181200

[b29] Ho A. & Dowdy S. F. Regulation of G(1) cell-cycle progression by oncogenes and tumor suppressor genes. Curr. Opin. Genet. Dev. 12(1), 47–52 (2002). 11790554 10.1016/s0959-437x(01)00263-5

[b30] Diaz-Moralli S., Tarrado-Castellarnau M., Miranda A. & Cascante M. Targeting cell cycle regulation in cancer therapy. Pharmacol. Ther. 138(2), 255–271 (2013). 23356980 10.1016/j.pharmthera.2013.01.011

[b31] Campbell K. J. & White R. J. MYC regulation of cell growth through control of transcription by RNA polymerases I and III. Cold. Spring Harb. Perspect Med. 4(5), doi: 10.1101/cshperspect.a018408. (2014). PMC3996375 24789877

[b32] Gabay M., Li Y. & Felsher D. W. MYC activation is a hallmark of cancer initiation and maintenance. Cold Spring Harb. Perspect. Med. 4(6), doi: 10.1101/cshperspect.a014241 (2014). PMC4031954 24890832

[b33] McMahon S. B. MYC and the control of apoptosis. Cold Spring Harb. Perspect Med. 4(7), a014407 (2014). 24985130 10.1101/cshperspect.a014407 PMC4066641

[b34] Dai M. S. . Inhibition of c-Myc activity by ribosomal protein L11. EMBO. J. 26(14), 3332–3345 (2007). 17599065 10.1038/sj.emboj.7601776 PMC1933407

[b35] Wu J. Y. . Reversal of multidrug resistance in cancer cells by pyranocoumarins isolated from Radix Peucedani. Eur. J. Pharmacol. 473(1), 9–17 (2003). 12877932 10.1016/s0014-2999(03)01946-0

[b36] Jung J. H. . Melatonin Suppresses the Expression of 45S Preribosomal RNA and Upstream Binding Factor and Enhances the Antitumor Activity of Puromycin in MDA-MB-231 Breast Cancer Cells. Evid. Based Complement Alternat. Med. 2013, 879746 (2013). 23690862 10.1155/2013/879746 PMC3638601

[b37] Ghosh A. . A new sesquiterpenoid coumarin from Ferula assafoetida. Nat. Prod. Commun. 4(8), 1023–1024 (2009). 19768976

